# Post-reflux swallow-induced peristaltic wave (PSPW): physiology, triggering factors and role in reflux clearance in healthy subjects

**DOI:** 10.1007/s00535-020-01732-5

**Published:** 2020-09-29

**Authors:** Mengyu Zhang, Busra Yaman, Sabine Roman, Edoardo Savarino, C. Prakash Gyawali, Jerry D. Gardner, Daniel Sifrim

**Affiliations:** 1 Wingate Institute of Neurogastroenterology, Blizard Institute, Barts and The London School of Medicine and Dentistry, Queen Mary University of London, 26 Ashfield Street, London, E1 2AJ UK; 2 Digestive Physiology, Hospital E Herriot, Hospices Civils de Lyon, Université de Lyon, Lyon, France; 3 Department of Surgery, Oncology and Gastroenterology, University of Padua, Padua, Italy; 4 Division of Gastroenterology, Washington University School of Medicine, St. Louis, MO USA; 5 Department of Gastroenterology, The First Affiliated Hospital of Sun Yat-sen University, Guangzhou, China; 6 Science for Organizations, Inc, Mill Valley, CA USA

**Keywords:** Gastroesophageal reflux, Esophageal clearance, Esophageal peristalsis

## Abstract

**Background and aims:**

The underlying physiology of post-reflux swallow-induced peristaltic wave (PSPW) is unclear. We aimed to: 1) calculate the probability of a random association between reflux and PSPW; 2) characterize factors that could underlie triggering of PSPW and 3) assess the chemical clearance effect of PSPW in healthy asymptomatic subjects.

**Methods:**

A total of 251 impedance–pH tracings from healthy asymptomatic subjects were analysed. Twenty consecutive tracings from this pool with 20–40 reflux episodes/24 h and a PSPW index higher than 50% were separately analyzed to evaluate the probability of a random association between reflux and PSPW. The characteristics of reflux episodes followed by a PSPW were compared with those not associated with PSPW.

**Results:**

A mean time interval of 29.3 s between a reflux episode and the first swallow captured 71% of total reflux episodes, and 67% of accompanying swallows were non-random. Compared to reflux without PSPW, reflux episodes with PSPW were more frequently acidic (*P* = 0.048), mixed with gas (*P* < 0.0001), of high proximal extent (*P* < 0.0001), while awake (*P* < 0.0001), and with shorter chemical clearance time (*P* = 0.040). High proximal extent, gas presence and occurring while awake were independent factors associated with PSPW (*P* < 0.0001).

**Conclusion:**

Using a time window between reflux and PSPW of around 30 s, the probability of a chance association is around 30%. Reflux episodes with high proximal extent, containing gas and occurring while awake are important factors associated with PSPW in healthy subjects. Reflux episodes with PSPW have a shorter chemical clearance time.

## Introduction

**Fig. 1:**
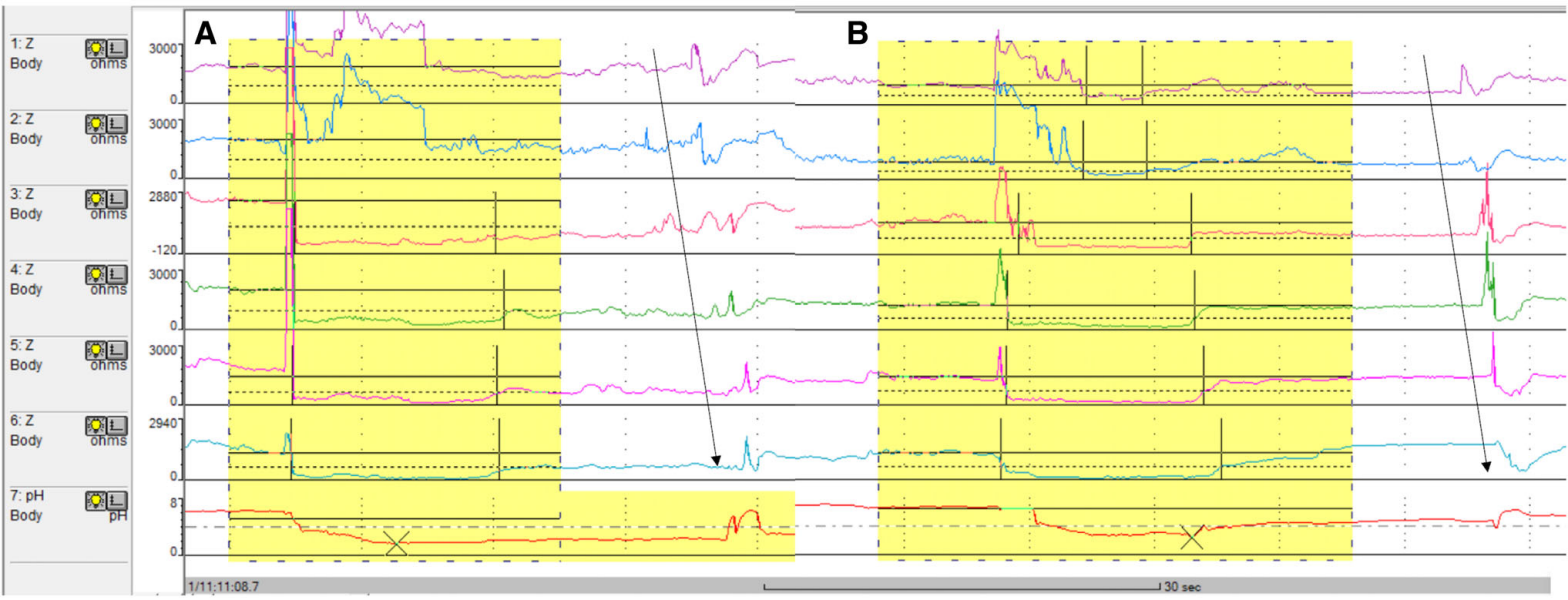
Reflux episodes and post-reflux swallow-induced peristaltic wave (PSPW) on pH–impedance monitoring. Reflux episodes are identified by at least 50% drop in impedance values from baseline, lasting at least 4 s in the distal two impedance channels, with retrograde propagation. In the two examples, acid reflux episodes contain gas and reach high proximal extent. Chemical clearance is observed after a swallow-induced peristaltic wave (PSPW), At least 50% drop in impedance is essential in the distal-most channel indicating arrival of neutralizing saliva at this location; correction of pH is observed in temporal relationship to the PSPW

Esophageal clearance of refluxed material is believed to be a major pre-epithelial defense mechanism against the development of gastroesophageal reflux disease (GERD) [1]. Reflux episodes are typically cleared via a two-step process, initial volume clearance, followed by chemical clearance [2]. Immediate swallow-induced primary peristalsis, or secondary peristalsis triggered by a distension-induced local reflex through activation of esophageal mechano-receptors, rapidly clears the bulk of the intraluminal refluxate volume, although remaining residue can sustain acidification of the esophageal mucosa. Chemical clearance of this residue involves a post-reflux swallow-induced peristaltic wave (PSPW), which delivers salivary bicarbonate and epidermal growth factor to the distal esophageal lumen for acid neutralization and potential repair of mucosal damage (Fig. 1). Experimental esophageal acidification and measurement of saliva production suggests that the swallow following acid reflux is elicited by an esophago-salivary reflex mediated through vagal afferents [3].

Esophageal impedance–pH metry can identify both acid and non-acid reflux episodes, with precise timing of reflux onset and volume clearance of the refluxate [4]. The pH metry component can determine the time taken for return to baseline esophageal pH following an acid reflux episode, which is a measure of chemical clearance. The impedance component can identify and quantify PSPW [5], from which the PSPW index is calculated as the ratio of reflux episodes followed by a PSPW to the total number of reflux episodes [6]. The PSPW index is reported to be lower in patients with GERD than in normal subjects or patients with functional esophageal disorders [6], with potential value as a diagnostic tool when GERD diagnosis is inconclusive [7] and as a relevant metric when mechanisms of refractoriness to proton pump inhibitor (PPI) therapy are evaluated [8–10].

Despite potential clinical relevance in GERD diagnosis and management, physiology underlying PSPW remains unclear, particularly, the mechanism for physiologic triggering of this reflex. Even in healthy subjects, only around 50% of reflux events are followed by a PSPW [11]. This raises two important questions. First, could a PSPW co-occur following a reflux episode simply by chance? Second, if PSPW is triggered by an esophagosalivary reflex [12], why do only certain reflux episodes trigger a PSPW while others do not, even in the same subject?

We hypothesized that systematic manual analysis of all reflux episodes (with and without associated PSPW) in a large series of impedance–pH tracings obtained from healthy asymptomatic subjects would elucidate the triggering mechanisms underlying PSPW. Thus, the aims of our study were: 1) to determine the probability of random association between reflux events and PSPW; 2) to characterize reflux-related factors underlying PSPW triggering, and 3) to assess the chemical clearance effect of PSPW in healthy asymptomatic subjects.

## Methods

### Subjects

Impedance–pH recordings performed using the Diversatek system (Boulder, Colorado, USA) were extracted from a multicenter international database of impedance–pH tracings obtained from healthy asymptomatic subjects from Asia, Europe, North America, and Latin America, and were included for analysis [13]. Exclusion criteria consisted of thoracic or digestive foregut surgery (except appendectomy), alcohol consumption > 40 g/d, use of medications that alter intra-gastric acidity or esophageal motility, as well as history of diabetes mellitus, neurologic disorders or other chronic gastrointestinal disease. Since the present study consists of post hoc analysis of previously collected de-identified pH–impedance data with no links to the original study subjects, Institutional Review Board approval was not deemed necessary.

### Ambulatory 24-h impedance–pH monitoring

A similar testing protocol (with variations in start time and meals) was followed by all international contributors of impedance–pH tracings. After at least 6 h fasting, the catheter was placed trans-nasally with one pH electrode positioned 5 cm proximal to the upper border of the LES, and six impedance channels with their mid-point located 3, 5, 7, 9,15, and 17 cm proximal to the LES. Subjects were encouraged to continue with their usual daily activities and meals during the recording period.

### Data analysis

Impedance–pH tracings were initially manually analysed by two expert reviewers working together either in-person or through video-conference using Bioview Analysis (Diversatek, Boulder, Colorado, USA). The reviewers, by consensus, excluded meal/drink induced pH drops and artefacts based on the impedance pattern, i.e. meals/drink are associated to very high swallowing frequency and prolonged low baseline impedance in distal channels, coinciding with the period of high-frequency swallowing. Thereafter, reviewers identified impedance reflux episodes and PSPW according to criteria established at a recent expert consensus meeting (Table 1).

**Table 1:** The Wingate Consensus recommendations for identification of reflux episodes and PSPW

Reflux episodes	PSPW
Meal times need to be correctly identified and excluded prior to evaluation of pH-impedance events	PSPW starts within 30 s after impedance returns to baseline in the distal most impedance channel following a reflux episode
A reflux episode consists of a 50% drop in impedance lasting for at least 4 s each in distal two impedance channels with retrograde propagation	PSPW does not need to be seen in all impedance channels as long as a swallow is identified in the most proximal channel with anterograde propagation in the proximal and distal-most impedance channels
A pH drop below 4.0 concurrent with a 4 s retrograde 50% impedance drop following a belch episode is counted as a reflux episode	An impedance drop of at least 50% below baseline needs to be present in the distal-most impedance channel
A pH drop without impedance detected reflux episode is counted as part of acid exposure time if not an artifact, but not as a reflux episode	Recovery of pH with antegrade impedance event is not mandatory but supports identification of PSPW
Automated analysis is first deployed, followed by manual addition of missed events and confirmation/deletion of identified reflux episodes using above criteria	PSPW is best evaluated using a 2 min window, using a 3000 ohms impedance scale

*PSPW* post-reflux swallow-induced peristaltic wave

A PSPW was defined as a swallow occurring within 30 s after the end of a reflux episode, provoking an antegrade 50% drop in impedance relative to the pre-swallow baseline originating in the most proximal impedance site, reaching all the distal impedance sites (Fig. 1). For each impedance–pH tracing, the number of reflux episodes followed within 30 s by a PSPW was divided by the total number of reflux episodes to obtain the PSPW index.

#### Probability of random association between reflux and PSPW

To evaluate the probability of random association between reflux and PSPW, we separately evaluated 20 consecutive tracings out of the total pool with approximately 20–40 reflux episodes/24 h and a PSPW index higher than 50%. In each impedance–pH tracing, meal periods were excluded and the following parameters were measured: the duration of the recording (in seconds), the total number of swallows, the total number of reflux episodes, and the time interval (in seconds) between the end of each reflux episode (determined as impedance recovery in most distal channel) and the next swallow (detected by impedance in the most proximal channel).

For each time interval between a reflux episode and the next swallow, the maximal possible number of such intervals was calculated by dividing the total recording time by the interval time.

For example, if the duration of the interval is 10 s and the duration of the recording is 24 h (86,400 s), there are 86,400/10 = 8,640 10-s intervals in the tracing. The probability that the occurrence of the swallow was random was calculated by dividing the total number of swallows during the recording by the maximal possible number of intervals. For example, if there are 1000 swallows, the probability of a swallow occurring in a 10-s interval by chance alone is 1000/8640 = 0.116.

The probability that the occurrence of swallows was random was calculated as follows:

Time interval between reflux and next swallow (seconds) × number of swallows duration of the recording (seconds).

For each time interval, we also calculated the fraction of the total number of reflux episodes that could be detected by this interval. For example, if for a given subject, the shortest interval between a reflux episode and a swallow is 5 s, a 5-s interval will detect one reflux episode. If the next longest interval between a reflux episode and a swallow, is 10 s, this interval will detect the swallow associated with this interval plus the swallow that was associated with the 5-s interval. This calculation is repeated up to the longest interval, which will capture all reflux episodes for the subject. The fraction of the total number of reflux episodes that could be detected by each interval is multiplied by one minus the probability that the accompanying swallow was random. The maximal value for this product is given for each subject in the last column of Table 2. Thus, for each subject, this maximal value identifies the time interval that captures the optimal number of reflux episodes associated with the highest probability of a non-random swallow.

**Table 2:** Time interval that captures the optimal number of reflux episodes associated with the highest probability of a non-random swallow from 20 asymptomatic subjects

Subject	Optimal interval (seconds)	Probability associated swallow is random	Probability associated swallow is not random	Reflux episodes detected (fraction total)	Maximal reflux episodes associated with non-random swallows
1	23.4	0.442	0.558	0.571	0.319
2	33.4	0.601	0.399	0.732	0.292
3	50.7	0.307	0.693	0.895	0.620
4	15.9	0.129	0.871	0.704	0.613
5	36.1	0.406	0.594	0.676	0.402
6	15.9	0.390	0.610	0.556	0.339
7	36.4	0.294	0.706	0.833	0.589
8	56.5	0.338	0.662	0.895	0.592
9	33.7	0.335	0.730	0.697	0.464
10	15.4	0.213	0.787	0.700	0.551
11	28.8	0.332	0.668	0.703	0.469
12	23	0.287	0.713	0.645	0.460
13	34.3	0.544	0.456	0.500	0.228
14	32.4	0.503	0.497	0.714	0.355
15	28.7	0.319	0.681	0.839	0.571
16	25.5	0.257	0.743	0.556	0.413
17	17.3	0.205	0.795	0.619	0.492
18	25.6	0.287	0.713	0.750	0.535
19	33.6	0.261	0.739	0.810	0.599
20	18.7	0.185	0.815	0.826	0.673
Mean (95% CI)	29.3 (27.6–30.9)	0.33 (0.31–0.35)	0.67 (0.65–0.69)	0.71 (0.69–0.73)	0.48 (0.46–0.50)

Data were normally distributed by the Kolmogorov–Smirnov test

*CI* confidence interval

#### Associated factors that might trigger PSPW and chemical clearance effect

For each of the impedance–pH tracings subjected to consensus expert analysis, the acid exposure time (total, upright and supine), characteristics of reflux episodes, and the PSPW index were extracted. The total acid exposure time was defined as the percentage of total recording time that the pH was below 4.

Seven characteristics were assessed for each reflux episode (with and without associated PSPW) as follows: (1) liquid and gas components of refluxate: liquid reflux was defined as a retrograde 50% drop in impedance in at least the two most distal impedance channels for ≥ 4 s, and gas reflux was defined as rapid impedance increase (> 3000 Ω/s) moving retrograde in at least two consecutive impedance segments. Reflux including gas (mixed reflux) was defined when gas reflux occurred immediately before or during liquid reflux; (2) nadir pH value: reflux episodes were classified as either acid (nadir pH < 4) or non-acid (nadir pH above 4); (3) pH change from baseline before reflux to nadir pH during reflux; (4) maximum pH recovery: the pH value reached after a PSPW; (5) proximal extent of reflux: high proximal extent was considered when liquid refluxate reached at least 15 cm above the LES; (6) acid clearance time: defined as the time in seconds from esophageal pH drop below 4 until recovery to a value of 4 or until a new reflux episode started. If the pH was already below 4 at the start of the reflux episode, a further pH drop of 1 pH unit indicated the start. (7) state of consciousness: whether the subject was awake or asleep at the time of the reflux episode: (identified by the combination of supine position and very low swallowing frequency of no more than one swallow every 3 min) [14].

### Statistical analysis

Continuous variables are presented as median (interquartile range, IQR), while categorical variables are presented as number (percentage). Comparisons between reflux episodes with and without PSPW were performed on a per subject basis using the Wilcoxon signed rank tests. Categorical data were compared using the $\chi^{2}$ test. To allow for multiple testing within the same dataset, a Bonferroni adjustment was used for p values from pairwise comparisons. Correlation between continuous variables was tested using Spearman’s rank correlation test. A stepwise approach retaining only significant variables with a *P* value of < 0.20 in univariate analysis was entered into a multivariate logistic regression model to identify predictors of triggering of PSPW in healthy subjects. The odds ratio (OR) and 95% confidence interval (CI) were calculated. All statistical analyses were conducted using SPSS 22.0 (SPSS Inc., Chicago, IL, USA). A *P* value of < 0.05 was considered to be statistically significant.

## Results

### Subjects

A total of 251 impedance–pH metry tracings from healthy asymptomatic subjects (median age 26 years, range 19–71 years, 57.4% female) were included for analysis, of which 33% were from Asia, 46% from Europe, 15% from North America, and 6% from Latin America. From within this pool, 20 consecutive tracings (median age 24.5 years, range 19–50 years, 25% female) were evaluated for probability of random association between reflux episodes and PSPW.

#### Probability of random association between reflux episodes and PSPW

The study duration of the 20 impedance–pH tracings included in this analysis ranged from 18.7 to 26 h (mean = 22 h); total reflux episodes ranged from 19 to 41 episodes (mean = 29); and total swallows ranged from 455 to 1907 (mean = 956). There was no significant correlation between study duration and number of reflux episodes (Spearman r = 0.106; *P* = 0.656) or number of swallows (Spearman *r* = 0.277; *P* = 0.237). There was, however, a significant correlation between number of reflux episodes and number of swallows (Spearman *r* = 0.726; *P* = 0.0003).

Table 2 demonstrates the time interval that captures the highest number of reflux episodes with the highest probability that the swallow following reflux episodes is non-random. The optimal time interval ranged from 15.4 to 56.5 s (mean = 29.3 s); the fraction of the total reflux episodes captured by the time interval ranged from 0.50 to 0.89 (mean = 0.71); and the probability that the swallow that occurred during the time interval was non-random ranged from 0.40 to 0.87 (mean = 0.67). These data showed that a mean time interval between a reflux episode and the first swallow of 29.3 s captured 71% of the total reflux episodes, and 67% of the accompanying swallows were non-random.

#### Factors involved in triggering of PSPW

##### Reflux episodes

In total, 6036 reflux episodes were detected and analysed, of which 3647 (60.4%) were acidic, and 3679 (61.0%) were mixed liquid–gas. A total of 1914 (31.7%) reflux episodes reached at least 15 cm above the LES. Most reflux episodes occurred during the awake period (96.7%).

On a per-subject basis, 21 (10, 35) reflux episodes were analyzed, of which 55.5% (33, 77) were acidic and 63.5% (47, 79) were mixed liquid–gas. The median acid exposure time was 0.4% (0.1–1.3). One quarter of reflux episodes reached at least 15 cm above the LES [25.0% (10, 41)], and 100% (96, 100) took place during the awake period (Table 3).

**Table 3:** Impedance–pH results from 251 asymptomatic subjects

Number of reflux episodes (*n*)		21 (10, 35)	
Number of PSPWs (*n*)		8 (4, 16)	
PSPW index (%)		48.0% (31, 60)	
The acidity (*n*, %)	Acid	10 (4, 23)	55.5% (33, 77)
	Non-acid	7 (4, 13)	45.5% (23, 67)
The presence of gas (*n*, %)	Mixed	12 (6, 21)	63.5% (47, 79)
	Liquid	7 (2, 15)	37.0% (21, 53)
Proximal extent (*n*, %)	High	5 (2, 11)	25.0% (10, 41)
	Low	13 (7, 24)	75.0% (59, 90)
Consciousness state at the time of reflux (*n*, %)	Awake	20 (10, 35)	100% (96, 100)
	Asleep	0 (0, 1)	0.0% (0, 4)
Acid exposure time (%)	Total	0.4 (0.1, 1.3)	
	Upright	0.6 (0.1, 1.5)	
	Supine	0.0 (0.0, 0.1)	

Data are presented as number (%) or median (interquartile range)

*PSPW* post-reflux swallow induced peristaltic wave.

##### PSPW

In total, 2753 reflux episodes (45.6%) were associated with PSPW while 3283 reflux episodes (54.4%) were not. A median of 8 (4, 16) PSPWs events were identified per subject. The median PSPW index was 48.0% (31, 60). No significant difference of PSPW index was found between male and females [48.0% (29, 61) vs. 48.0% (32, 60), *P* = 0.922]. There was no significant correlation between age (median age 26 years, range 19–71 years) and PSPW index (*r* = − 0.031, *P* = 0.630).

##### Comparison between reflux episodes with and without PSPW

**Fig. 2:**
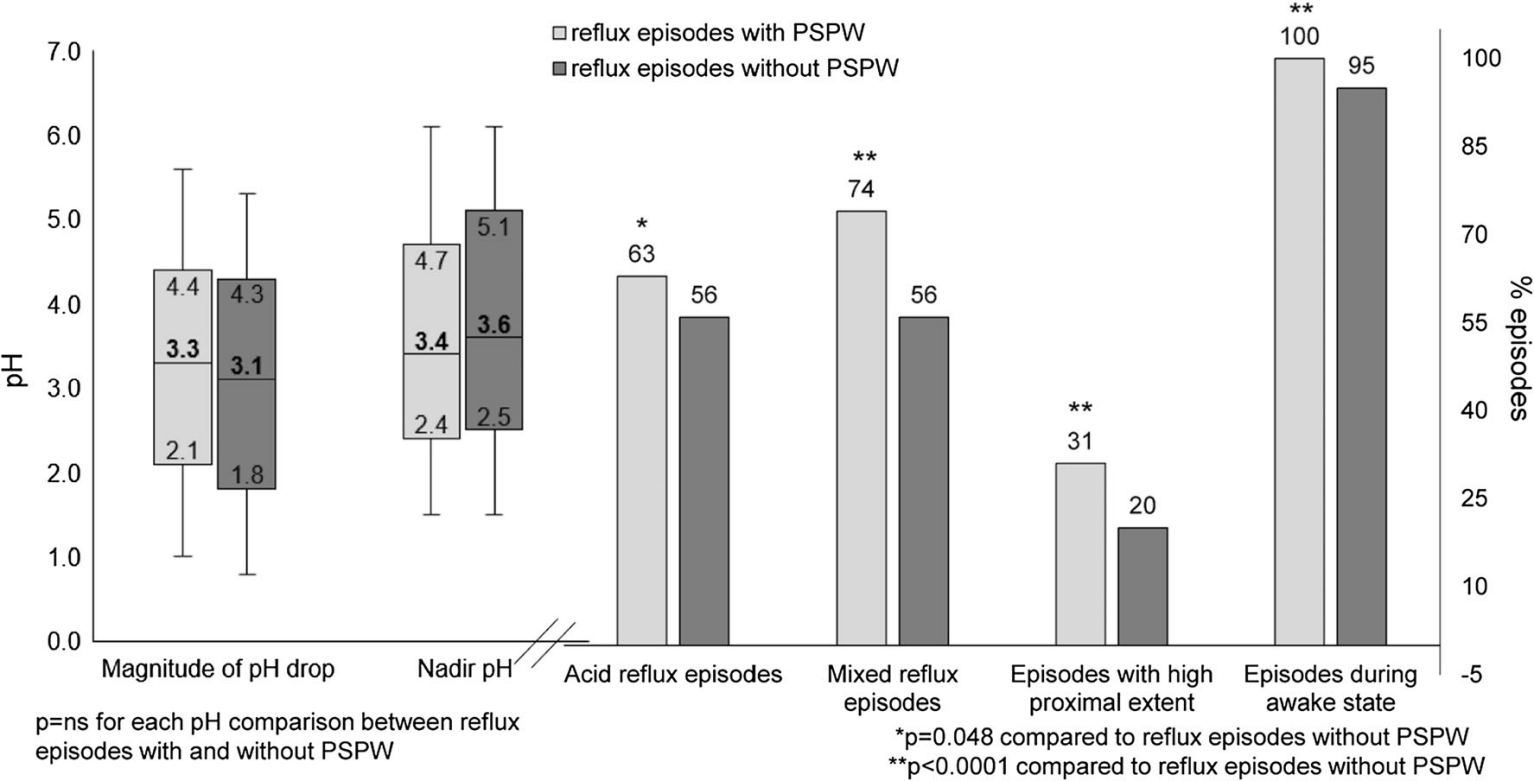
Comparison between reflux PSPW ( +) versus reflux PSPW (−) from 251 asymptomatic subjects. There was no significant difference in magnitude of pH drop and nadir pH. However, reflux PSPW ( +) episodes were more frequently acid, containing gas and reaching high proximal extent and occurred in the awake state. Data are shown as box and whiskers plot on the left (box showing median and interquartile range and whiskers showing 5 and 95 percentiles) and as median values on the right

The magnitude of the pH drop was larger and the nadir pH was lower in the reflux episodes with PSPW compared to without PSPW, but these did not reach statistical significance (Fig. 2). However, reflux episodes with PSPW were acidic significantly more often than reflux episodes without PSPW (63.0% vs. 56.0% respectively, *P* = 0.048). Besides, compared to reflux episodes without PSPW, presence of PSPW was associated more often with mixed reflux with gas (74.0% vs. 56.0%, *P* < 0.0001), and reached higher proximal extent (*P* < 0.0001). There was a higher proportion of reflux episodes reaching at least 15 cm above the LES when PSPW was present than when absent (31.0% vs. 20.0%, *P* < 0.0001). Reflux episodes with PSPW occurred significantly more often in the awake state (100% vs. 95.0%, *P* < 0.0001).

##### Factors associated with PSPW

Univariate and multivariate analysis showed that presence of gas in the refluxate, high proximal extent of reflux and awake state were independent associated factors that might be involved in triggering of PSPW (Table 4, Fig. 1).

**Table 4:** Univariate and multivariate regression analysis of associated factors that might trigger PSPW from 251 asymptomatic subjects

	Univariate analysis			Multivariate analysis	
	Odds, reflux with PSPW vs. odds, reflux without PSPW	Odds ratio (95%CI)	*P* value	Odds ratio (95%CI)	*P* value
The acidity (acid vs. non-acid)	1.662 vs. 1.423	1.168 (1.053–1.296)	0.003		
The presence of gas (mixed vs. liquid)	2.136 vs. 1.220	1.751 (1.575–1.946)	< 0.0001	1.630 (1.464–1.816)	< 0.0001
Proximal extent (high vs. low)	0.603 vs. 0.365	1.653 (1.482–1.843)	< 0.0001	1.588 (1.422–1.773)	< 0.0001
Consciousness state (awake vs. asleep)	61.568 vs. 20.044	3.072 (2.190–4.309)	< 0.0001	2.326 (1.648–3.282)	< 0.0001

The column entitled “Odds, reflux with PSPW vs. odds, reflux without PSPW” compares reflux episodes with and without PSPW in terms of Odds ratios

*PSPW* post reflux swallow induced peristaltic wave. *CI* confidence interval

#### Effect of PSPW in chemical clearance of reflux episodes

When PSPW followed acidic reflux episodes (1719 episodes), the PSPW increased distal esophageal pH from below to above pH 4 in 1603 episodes (93.3%). The pH value increased significantly after PSPW from 2.3 (1.6, 3.0) to 6.2 (5.3, 6.9) (*P* < 0.0001). The acid clearance time was significantly shorter in the acidic reflux episodes with PSPW compared to acidic reflux episodes without PSPW [16.1 s (9.1, 30.1) vs. 19.0 s (9.5, 46.7), *P* = 0.040]. In 1034 non-acidic reflux episodes which were associated with PSPW, the pH value also increased significantly after PSPW [5.5 (4.7, 6.3) to 7.0 (6.5, 7.5), *P* < 0.0001].

## Discussion

Post-reflux swallow-induced peristaltic wave (PSPW), and PSPW index are recently described metrics on impedance–pH metry relevant to GERD diagnosis and management [5], because values are lower in GERD compared to normal subjects and functional esophageal disorders [6]. Since physiology underlying PSPW remains incompletely understood, we evaluated a large number of impedance–pH tracings obtained from healthy asymptomatic subjects, to better understand relationships of PSPW to reflux episodes, triggering factors, and chemical clearance effect. We report that the calculated probability of a chance association between reflux episodes and PSPW is no more than 30% when a time window of around 30 s is used for PSPW definition. Furthermore, reflux episodes with high proximal extent, containing gas, and occurring in the awake state are more likely to be associated with a PSPW. More importantly, we found that reflux episodes followed by a PSPW have a shorter chemical clearance time than those without, highlighting the physiological significance of this entity.

The description of PSPW based on a time relationship between a swallow following a previous reflux episode allows for several physiological possibilities including a chance association, a neural mediated reflex or a conscious swallow after reflux perception. Considering that a healthy subject swallows around 900 times a day (excluding meals) and has around 30 reflux episodes, the probability of a random association is relevant. Our analysis of 20 impedance–pH tracings with adequate reflux and PSPW events was performed to identify the optimal time window between the events to preclude the chance of random association. Interestingly, a time window of 29 s between reflux and PSPW (almost identical to the currently utilized 30 s time window) was associated with the lowest probability of random association. It is important to consider that there remains a 30% of probability of random association even with this optimal time window. However, against the concept of random association is the lower PSPW index in GERD, especially when poorly responsive to PPI, compared to healthy subjects [6, 8–11]. Based on our analysis, the time window for PSPW identification can potentially be adjusted according to the purpose of PSPW identification, i.e. to increase sensitivity (longer window) vs. specificity (shorter window) for diagnosis of GERD, since this will modify the chance of random association.

We found a statistically significant linear relationship between number of swallows and number of reflux events. This is the first time that such relationship is described and required manual counting of all swallows during the recordings. This correlation might imply that reflux triggers more swallows, or on the contrary, swallows allow for more reflux to occur. Further investigation is required to clarify this relationship between swallows and reflux.

Several factors contribute to the understanding of PSPW as a reflex triggered by reflux episodes. Healthy subjects swallow around once/minute [15] and the latency period of salivary glands to secrete saliva in response to esophageal acidification is reported to be 10–15 s [3]. A time window of 30-s post-reflux was considered optimal for PSPW identification to account for the swallowing frequency and the latency of salivary secretion to experimental esophageal acidification [12]. The esophago-salivary reflex can be inhibited by anesthetization of either the lower esophagus or salivary glands using lidocaine [3], which blocks the sensory fibers responsible for reflex activity [16, 17]. Our study amongst healthy subjects showed that only 45.6% of reflux episodes were followed by a PSPW. Asymptomatic healthy subjects are expected to have the most robust reflex. It is unclear why only half of reflux episodes trigger this “reflex”, and further investigation is warranted.

The parameters of the regression equation also provide insight into possible physiologic relationships between reflux episodes and swallows. Nearly all previous analyses of possible relationships between reflux episodes and swallows, including the present one, have examined only the first swallow that follows a reflux episode and, in many instances, only the swallow that occurs within 30 s of a reflux episode. It remains plausible that terminating signals initiated by the reflux episode and accompanying esophageal acidification is sufficiently important from the physiologic standpoint that more than one swallow is recruited to neutralize the reflux episode.

The possibility of PSPW being a conscious response to reflux perception remains to be formally investigated. It is worth noting that factors triggering PSPW in this study (high proximal extent of reflux, gas containing reflux, reflux while awake) are the same factors reported in the perception of GERD symptoms [18, 19]. However, it is unlikely that these factors work similarly when triggering PSPW vs. symptoms. Patients with esophageal hypersensitivity, manifest as typical reflux symptoms like heartburn or chest pain in the absence of pathological reflux (reflux hypersensitivity and functional heartburn) have opposite PSPW index values compared to pathologic GERD [6, 8]. On the contrary, if PSPW is triggered by conscious perception in healthy subjects, it should be related to subtle perception of reflux, since the subjects are asymptomatic by definition. In favor of this, we demonstrate that PSPW occurred mainly in the awake state, with reflux episodes with high proximal extent and presence of gas.

Our results suggest that reflux events containing gas, with lower nadir pH and higher proximal extent may result in a greater mechanical (distension) and chemical (acid) stimulation of afferent nerves involved either in reflex or conscious perception. If this is indeed the case, the stimulus seems to impact both the distal and proximal esophagus. Our group has recently described the human sensory esophageal mucosal innervation in detail. Proximal esophageal innervation is more superficial than in the distal esophagus, suggesting that the proximal esophagus is more sensitive, and thereby prone to trigger reflexes when stimulated [20].

Based on our findings and supporting literature, it is possible to speculate mechanisms underlying impaired triggering of PSPW among patients with GERD. First, pure liquid reflux might be more frequent in more severe GERD patients, thereby reducing triggering of PSPW. Second, while acidity of refluxate appears to influence triggering of PSPW in healthy subjects, GERD patients have impaired chemical clearance in the setting of significantly more acid reflux [21]. Therefore, acid may play a less crucial role in the triggering of PSPW in GERD patients, as demonstrated by a recent study [22]. Third, high proximal extent of reflux, another important triggering factor of PSPW, leads to another paradox where GERD patients have significantly higher proximal extent of both acid and non-acid reflux, but lower PSPW index compared to normal subjects [21, 23]. Moreover, patients with GERD have significantly more nocturnal reflux episodes than control subjects [24, 25], and PSPW is scarce during sleep as a result of a low swallowing rate and infrequent peristalsis [26, 27]. The lower PSPW index in GERD patients could be a consequence of their mucosal disease, or vice versa, i.e. the lack of adequate chemical clearance mechanisms contributes to mucosal damage [28, 29]. Finally, rather than impaired triggering of PSPW, GERD patients could have normal numbers of PSPW events, with a reduced PSPW index simply because of a higher number of total reflux episodes. The calculation of the PSPW index in the presence of multiple successive reflux episodes is a limitation of this parameter. The same limitation occurs when calculating SAP. In the current study, healthy subjects did not show multiple successive reflux episodes. This is more commonly observed in patients with hiatal hernias or other mechanical disorders.

Interestingly, PSPW events were associated with not just acidic but also weakly acidic reflux episodes. Although chemical clearance involving PSPW was initially thought to be elicited by significant esophageal acidification, we found that 43.3% of weakly acid refluxes were associated with PSPW. This suggests that small variations in esophageal mucosal pH together with distension produced by non-acid reflux may trigger PSPW, not only to increase mucosal pH but also to bring mucosal protectants present in saliva to the distal esophagus. Gastroesophageal refluxate consists of noxious agents such as hydrochloric acid and pepsins, and the proteolytic activity of pepsins is maintained up to pH 6 [30]. Experimental work using esophageal perfusion have found that weakly acidic solutions also impair mucosal integrity [31]. Therefore, weakly acidic refluxes are also deleterious for the esophageal mucosa and can trigger protective PSPWs.

The limitations of this study should be acknowledged. The aim was to study PSPW as a physiological mechanism in humans. This is a retrospective review of impedance–pH tracings from asymptomatic healthy subjects. The refluxate factors involved in patients with GERD need to be assessed and compared with our findings in healthy subjects. We will also assess the predictive value of different time windows to calculate PSPW in terms of response to medical or surgical treatment in patients with GERD. Our calculation of the probability that a swallow is random assumes that swallows are uniformly distributed over the entire recording. Since swallows and reflux vary, and increase in frequency during various periods during the day, our calculations underestimate the probability that a swallow is random. In healthy subjects, the number of nocturnal reflux episodes during sleep was very limited. However, the observation that these nocturnal reflux episodes were not followed by PSPW was very consistent. This study did not assess reflux perception and its relationship with PSPW, and this will need to be investigated to understand the role of hypersensitivity or vigilance in PSPW triggering. Finally, we were unable to measure the volume of the refluxate which may also influence the triggering of PSPW.

In conclusion, despite the potential clinical relevance of PSPW in the diagnosis and management of GERD, the physiology underlying PSPW is complex and was not completely understood. In this study, we found that using a time window between reflux and PSPW of around 30 s, the probability of a chance association is no more than 30%. Reflux events with high proximal extent, containing gas and occurring in the awake state are more likely to be associated with a PSPW. Reflux events followed by a PSPW had a shorter chemical clearance time than those without a PSPW. The mechanism of impaired triggering of PSPW in patients with GERD still needs to be investigated.
